# Cardiovascular Outcomes and Mortality After Bariatric Surgery in Patients With Nonalcoholic Fatty Liver Disease and Obesity

**DOI:** 10.1001/jamanetworkopen.2023.7188

**Published:** 2023-04-07

**Authors:** Arunkumar Krishnan, Yousaf Hadi, Saleh A. Alqahtani, Tinsay A. Woreta, Wei Fang, Salim Abunnaja, Nova Szoka, Lawrence E. Tabone, Shyam Thakkar, Shailendra Singh

**Affiliations:** 1 Section of Gastroenterology and Hepatology, West Virginia University School of Medicine, Morgantown; 2 Division of Gastroenterology and Hepatology, Johns Hopkins University School of Medicine, Baltimore, Maryland; 3 West Virginia Clinical & Translational Science Institute, Morgantown; 4 WVU Medicine Center for Weight Management, Morgantown, West Virginia; 5 Department of Surgery, West Virginia University School of Medicine, Morgantown

## Abstract

**Importance:**

Bariatric surgery (BS) is associated with significantly reduced incidence of cardiovascular diseases and mortality in patients with obesity. However, whether BS can decrease major adverse cardiovascular events in patients with nonalcoholic fatty liver disease (NAFLD) remains poorly understood.

**Objective:**

To investigate the association of BS with the incidence of adverse cardiovascular events and all-cause mortality in patients with NAFLD and obesity.

**Design, Setting, and Participants:**

This was a large, population-based, retrospective cohort using data from the TriNetX platform. Adult patients with a body mass index (BMI, calculated as weight in kilograms divided by height in meters squared) of 35 or greater and NAFLD (without cirrhosis) who underwent BS between January 1, 2005, and December 31, 2021, were included. Patients in the BS group were matched with patients who did not undergo surgery (non-BS group) according to age, demographics, comorbidities, and medication by using 1:1 propensity matching. Patient follow-up ended on August 31, 2022, and data were analyzed in September 2022.

**Exposures:**

Bariatric surgery vs nonsurgical care.

**Main Outcomes and Measures:**

The primary outcomes were defined as the first incidence of new-onset heart failure (HF), composite cardiovascular events (unstable angina, myocardial infarction, or revascularization, including percutaneous coronary intervention or coronary artery bypass graft), composite cerebrovascular disease (ischemic or hemorrhagic stroke, cerebral infarction, transient ischemic attack, carotid intervention, or surgery), and a composite of coronary artery procedures or surgeries (coronary stenting, percutaneous coronary intervention, or coronary artery bypass). Cox proportional hazards models were used to estimate hazard ratios (HRs).

**Results:**

Of 152 394 eligible adults, 4693 individuals underwent BS; 4687 patients who underwent BS (mean [SD] age, 44.8 [11.6] years; 3822 [81.5%] female) were matched with 4687 individuals (mean [SD] age, 44.7 [13.2] years; 3883 [82.8%] years) who did not undergo BS. The BS group had significantly lower risk of new-onset of HF (HR, 0.60; 95% CI, 0.51-0.70), cardiovascular events (HR, 0.53; 95% CI, 0.44-0.65), cerebrovascular events (HR, 0.59; 95% CI, 0.51-0.69), and coronary artery interventions (HR, 0.47; 95% CI, 0.35-0.63) compared with the non-BS group. Similarly, all-cause mortality was substantially lower in the BS group (HR, 0.56; 95% CI, 0.42-0.74). These outcomes were consistent at follow-up duration of 1, 3, 5, and 7 years.

**Conclusions and Relevance:**

These findings suggest that BS was significantly associated with lower risk of major adverse cardiovascular events and all-cause mortality in patients with NAFLD and obesity.

## Key Points

**Question:**

Is bariatric surgery associated with the incidence of major adverse cardiovascular events or all-cause of mortality among patients with nonalcoholic fatty liver disease (NAFLD) and obesity?

**Findings:**

In this cohort study of 152 394 patients with NAFLD and obesity (including 4693 patients who underwent bariatric surgery and 147 701 patients in the nonsurgical control group), bariatric surgery was significantly associated with lower risks of major adverse cardiovascular events and all-cause mortality. These outcomes were consistent at follow-up durations of 1, 3, 5, and 7 years.

**Meaning:**

These findings suggest that bariatric surgery was associated with a substantially lower risk of incidents of major adverse cardiovascular events and all-cause mortality in patients with NAFLD and obesity.

## Teaser

This cohort study assesses the association of bariatric surgery with the incidence of adverse cardiovascular events and all-cause mortality in patients with nonalcoholic fatty liver disease (NAFLD) and obesity.

## Introduction

Nonalcoholic fatty liver disease (NAFLD) is one of the most common causes of liver disorders in the US and is the leading cause of chronic liver disease globally.^1^ The global prevalence of NAFLD has reached 25.2% and is projected to be 33.5% by 2030.^2^ NAFLD is considered a hepatic manifestation of metabolic syndrome, which is characterized as a cluster of metabolic disorders, such as obesity, insulin resistance, type 2 diabetes, hypertriglyceridemia, dyslipidemia, and hypertension, which are all risk factors associated with cardiovascular diseases (CVDs).^3^ Several cohort studies have steadily acknowledged that NAFLD is associated with a significantly higher risk of all-cause mortality and that the leading causes of death among patients with NAFLD are CVDs.^4^ The multifaceted nature of NAFLD with varying coexisting metabolic complications makes its treatment complex. Interventions that target NAFLD-associated obesity could potentially reduce the incidence of CVDs and mortality in this patient group. However, no specific pharmacotherapy is approved to treat NAFLD, and lifestyle change aimed at weight loss remains the mainstay of clinical management.^4,5^

Bariatric surgery (BS) is an efficient weight-loss intervention in patients with obesity. BS may be indicated in patients with obesity and NAFLD to achieve and maintain the degree of weight loss (ie, 7%-10%) associated with therapeutic outcomes. However, there is a paucity of data on the impact of BS specifically for patients with NAFLD, and the outcomes of BS in patients with obesity cannot be directly extrapolated for patients with NAFLD. The pathophysiology behind the association of NAFLD with CVD is not completely understood, and CVD development in NAFLD may involve other pathways besides obesity and insulin resistance alone, including low-grade inflammation, oxidative stress, and the effects of perturbations in the gut microbiota.^6,7^ It has been known that patients with NAFLD constitute a higher-risk cohort for CVD independent of body mass index (BMI; calculated as weight in kilograms divided by height in meters squared) and obesity.^7^ Thus, the association of BS with NAFLD warrants further study. Therefore, we aimed to explore the association of BS with major adverse cardiovascular events and all-cause mortality at a population level.

## Methods

This large, population-based, retrospective cohort study was conducted using TriNetX (Cambridge, Massachusetts), a federated health research network data set. TriNetX is a multi-institutional health research network that provides deidentified electronic health records (EHRs) from included health care organizations. Clinical variables are derived directly through EHRs. Robust quality assurance on the network is achieved at the time of extraction from the EHRs before inclusion in the data set. The platform only provides aggregate patient counts and statistical summaries to ensure deidentification at all levels of retrieval and dissemination of patient data. TriNetX received a waiver of review and informed consent from WIRB institutional review board as a federated network since it includes only aggregated counts and statistical summaries of deidentified information. Still, no protected health information was obtained, and no study-specific activities were performed in this retrospective analyses. Details of the data source are described in the eAppendix in Supplement 1. We followed the Strengthening the Reporting of Observational Studies in Epidemiology (STROBE) reporting guideline.

### Study Participants

All adult patients (aged >18 years) with a diagnosis of NAFLD and obesity with BMI of 35 or greater in the TriNetX database between January 1, 2005, and December 31, 2021, were identified. Follow-up of these patients ended on August 31, 2022. Patients were excluded if they met any of the following criteria: chronic liver disease other than NAFLD, including alcohol, viral, drug-induced, autoimmune, and genetic diseases, liver cirrhosis, or clinical diagnosis of hepatic decompensation (such as esophageal varices or ascites); BMI less than 35 (ineligible for bariatric surgery); history of excessive alcohol use, alcohol abuse, or alcohol use disorder or history of alcohol-related disorders; HIV infection; solid organ transplantation; and history of undergoing dialysis treatment. Finally, patients with a history of heart failure (HF), ischemic heart disease, unstable angina, myocardial infarction, aortic aneurysm or dissection, stroke (ischemic or hemorrhagic stroke), cerebral infarction, transient ischemic attack, carotid intervention or surgery, coronary stenting, percutaneous coronary intervention (PCI), or coronary artery bypass before inclusion to the cohort or prior to the index event were also excluded.

### Study

Patients who met the inclusion criteria were divided into 2 groups: those who underwent BS (study group) and those who did not undergo BS (control group). BS procedures included Roux-en-Y gastric bypass (RYGB) or sleeve gastrectomy (SG). Patients with a history of gastric banding and other less common bariatric procedures were excluded. All bariatric surgical procedures were defined using the diagnosis and current procedural terminology suggested by the American Society for Metabolic and Bariatric Surgery for RYGB or SG. Details of diagnosis and procedure codes used for patient selection are described in the eAppendix in Supplement 1.

BS was considered the index event for patients in the BS group. The index event for the control group was defined as the first time that the patient was eligible for inclusion in the study (based on a diagnosis of NAFLD and obesity with BMI ≥35) during the a priori–defined study time period (January 1, 2005, to December 31, 2021).

### Matching Process

Each patient in the BS group was matched to a patient in the non-BS group using 1:1 propensity score matching (PSM) to reduce confounding.^8^ Covariates in the propensity score model were adjusted for a priori–identified potential confounders: age, sex, self-reported race and ethnicity (Hispanic, non-Hispanic Black, non-Hispanic White, or non-Hispanic other [eg, Asian, American Indian or Alaska Native, Native Hawaiian, or other Pacific Islander]), nicotine dependence, BMI, diabetes, hypertension, hyperlipidemia, chronic respiratory disease, chronic kidney diseases, blood pressure, oral diabetes medication use, insulin use, use of angiotensin-converting enzyme inhibitor, β-blockers, antiarrhythmics, antilipemic agents, angiotensin receptor–blocker medications, use of any other antihypertensive medication, cholesterol level, low-density lipoprotein level, and serum triglyceride level. Race and ethnicity were included in analyses because of the associated differences in BS outcomes.^9^ Logistic regression on these input matrices was used to obtain propensity scores for each patient in both cohorts. Logistic regression was performed in Python software version 3.6.5 (Python Software Foundation) using standard libraries NumPy and sklearn. The same analyses were also performed in R software version 3.4.4 (R Project for Statistical Computing) to ensure outputs matched. After calculating propensity scores, matching was performed using a greedy nearest-neighbor matching algorithm with a caliper of 0.1 pooled SDs. The order of the rows in the covariate matrix can affect the nearest neighbor matching; therefore, the order of the rows in the matrix was randomized to eliminate this bias.

### Study Outcomes

The primary outcome of our study was to assess the incidence or new onset of major adverse cardiovascular events categorized as HF, composite cardiovascular events, composite cerebrovascular events, and composite coronary artery interventions. Composite cardiovascular events were defined as the first occurrence of unstable angina, myocardial infarction, or revascularization, including PCI or coronary artery bypass graft.^10,11^ Composite of cerebrovascular disease was defined as the first occurrence of stroke (ischemic or hemorrhagic stroke), cerebral infarction, transient ischemic attack, carotid intervention, or surgical procedure.^10,11^ The composite of coronary artery procedures or surgical treatments was based on the requirement for coronary stenting, PCI, or coronary artery bypass. The secondary outcome was to evaluate the incidence of all-cause mortality. Patients with CVD and mortality outcomes before the index period were excluded at the time of patient selection. Major surgical complications were estimated to assess the safety of BS in this patient population 30 days after BS.

### Statistical Analysis

All statistical analyses were performed in real time using the TriNetX platform. Continuous variables are expressed as mean and SD. Categorical variables are presented as frequency and percentage. Patients were matched using PSM, and balancing of potential confounding variables between the BS and the control group after PSM was evaluated using standardized mean differences (SMD) with a threshold set a priori at 0.10. SMD was used to measure the magnitude of difference between the groups rather than the *P* value because of the insensitivity of SMDs to sample size.^12^ For each outcome, Cox proportional hazards models were used to estimate the hazard ratios (HRs). HRs and 95% CIs, along with tests for proportionality, were calculated using the survival package in R version 3.2.3. HRs and 95% CIs adjusted for baseline variables were calculated and reported for all analyses. Numbers were then validated by comparing them with the output from SAS statistical software version 9.4 (SAS Institute). Kaplan-Meier survival analyses were also used to estimate the survival probability of the outcome at the end of 7 years after the index event. Patients were censored when the time window ended or on the day after the last event in their record. Hypothesis testing for Kaplan-Meier survival curves was conducted by using the log-rank test. A priori 2-sided α < .05 was used for statistical significance. Data were analyzed in September 2022.

#### Sensitivity Analysis

The benefits of BS may not be apparent immediately after the procedure, and any short-term outcomes in this population may be due to multiple chronic high-risk factors. Therefore, we performed a sensitivity analysis by estimating risk after excluding patients with outcomes within 1 year or 2 years after the index event.

#### Secondary Analysis

Secondary analysis was performed comparing CVD outcomes between a cohort of patients with obesity without NAFLD who underwent BS and patients with obesity without NAFLD who did not undergo BS. HRs were calculated, adjusted for the same confounders as the primary analyses.

## Results

**Figure 1.**
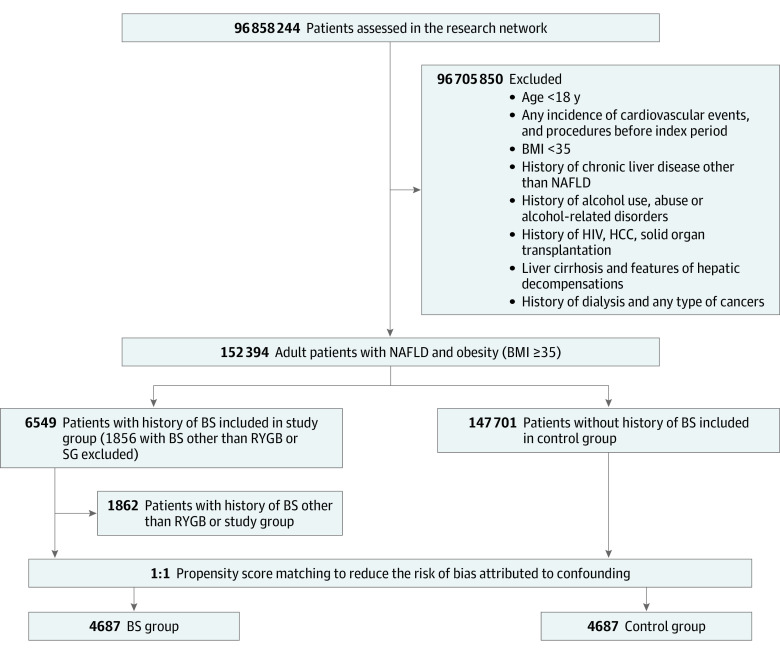
Flowchart of Selection of Patients With Obesity and Nonalcoholic Fatty Liver Disease (NAFLD) Who Underwent Bariatric Surgery (BS) and Who Did Not BMI indicates body mass index (calculated as weight in kilograms divided by height in meters squared); HCC, hepatocellular carcinoma; RYGB; roux-en-Y gastric bypass; and SG, sleeve gastrectomy.

A total of 152 394 patients with obesity and NAFLD were identified. Among these patients, 4693 had a BS history (BS group), and 147 701 did not have a BS history. Of the BS group, SG accounted for 65% of procedures, whereas the rest had a history of RYGB. (Figure 1). After PSM, a total of 4687 patients were included in each group (BS: mean [SD] age, 44.8 [11.6] years; 3822 [81.5%] female; control: mean [SD] age, 44.7 [13.2] years; 3883 [82.8%] female). The groups were well-matched after PSM (SMD, <0.1) (Table 1; eFigure 1 in Supplement 1). Only minor residual imbalances remained after PSM (SMD, <0.25).

**Table 1.** Baseline Characteristics of BS and Non-BS Group Patients With Obesity and Nonalcoholic Fatty Liver Disease

Characteristic	Patients, No. (%)		Standard mean difference
	BS (n = 4687)	Non-BS (n = 4687)	
Age, mean (SD), y	44.8 (11.6)	44.7 (13.2)	0.0040
Sex			
Female	3822 (81.5)	3883 (82.8)	0.0340
Male	865 (18.5)	804 (17.2)	
Race and ethnicity			
Hispanic or Latino	419 (8.9)	398 (8.4)	0.0159
Non-Hispanic Black	725 (15.4)	751 (16.0)	0.0152
Non-Hispanic White	3409 (72.7)	3431 (73.2)	0.0106
Non-Hispanic other^a^	498 (10.6)	460 (9.8)	0.0268
BMI, mean (SD)	43.4 (4.97)	42.2 (5.26)	0.2379^b^
Nicotine dependence	692 (14.7)	638 (13.6)	0.0330
Comorbidities			
Type 2 diabetes	1626 (34.6)	1592 (33.9)	0.0153
Hypertension	2732 (58.2)	2704 (57.6)	0.0121
Hyperlipidemia	1880 (40.1)	1842 (39.3)	0.0166
Obstructive sleep apnea	2407 (51.3)	2320 (49.4)	0.0371
Atrial fibrillation and flutter	139 (2.9)	137 (2.9)	0.0025
Chronic lower respiratory diseases	1538 (32.8)	1558 (33.2)	0.0091
Blood pressure, mean (SD), mm Hg			
Systolic	132 (16.7)	129 (16.0)	0.1964^b^
Diastolic	78.3 (10.4)	78.2 (11.0)	0.0079
HbA_1c_, %	6.15 (1.22)	6.48 (1.65)	0.2253^b^
Liver function panel, mean (SD)			
ALT, U/L	35.4 (31.5)	39.0 (37.8)	0.1042^b^
AST, U/L	27.8 (22.1)	30.1 (26.0)	0.0964
ALP, U/L	84.6 (27.9)	86.9 (34)	0.0767
Total bilirubin, mg/dL	0.469 (0.248)	0.49 (0.487)	0.0553
Albumin, g/dL	4.1 (0.394)	4.0 (0.485)	0.2208
Protein, g/dL	7.19 (0.597)	7.19 (0.599)	0.0072
Blood counts, mean (SD)			
Hemoglobin, g/dL	13.4 (1.49)	13.2 (1.71)	0.0974
Platelets, ×10^3^/µL	281 (72)	275 (73.4)	0.0807
Kidney function, mean (SD)			
Creatine, mg/dL	0.86 (0.46)	0.86 (1.18)	0.0002
BUN, mg/dL	14.4 (6.44)	13.6 (5.98)	0.1264^b^
Lipid panel, mean (SD)			
Cholesterol, mg/dL	180 (39.2)	181 (40.9)	0.0452
Triglyceride, mg/dL	163 (117)	168 (134)	0.0381
HDL, mg/dL	45.5 (12.3)	45.1 (12.5)	0.0287
LDL, mg/dL	103 (33.0)	105 (33.8)	0.0390
Coagulation profile, mean (SD)			
PT, s	12.5 (4.34)	12.6 (4.08)	0.0355
INR	1.12 (0.688)	1.18 (2.02)	0.0386
Medications			
Cardiovascular			
Antiarrhythmics	2164 (46.1)	2104 (44.8)	0.0257
β-Blockers	1227 (26.1)	1155 (24.6)	0.0353
Antilipemic agents	1236 (26.3)	1161 (24.7)	0.0367
ACE inhibitors	1071 (22.8)	949 (20.2)	0.0633
Angiotensin-II inhibitor	614 (13.1)	585 (12.4)	0.0185
Antihypertensives	509 (10.8)	480 (10.2)	0.0201
Antidiabetic			
Oral hypoglycemic agents	1219 (26.0)	1239 (26.4)	0.0097
Insulin	666 (14.2)	760 (16.2)	0.0559
Supplements			
Vitamin D	1539 (32.8)	1103 (23.5)	0.2079^b^
Vitamin E	195 (4.1)	129 (2.7)	0.0771

Abbreviations: ACE, angiotensin-converting enzyme; ALP, alkaline phosphatase; ALT, alanine aminotransferases; AST, aspartate aminotransferase; BMI, body mass index (calculated as weight in kilograms divided by height in meters squared); BS, bariatric surgery; BUN, blood urea nitrogen; HbA_1c_, hemoglobin A_1c_; HDL, high-density lipoprotein; INR, international normalized ratio; LDL, low-density lipoprotein; PT, prothrombin time.

SI conversion factors: To convert albumin to grams per liter, multiply by 10; ALP, ALT, and AST to microkatals per liter, multiply by 0.0167; BUN to millimoles per liter, multiply by 0.357; cholesterol measures to millimoles per liter, multiply by 0.0259; creatine to micromoles per liter, multiply by 76.25; HbA_1c_ to proportion of total hemoglobin, multiply by 0.01; hemoglobin to grams per liter, multiply by 10; platelets to ×10^9^/L, multiply by 1; protein to grams per liter, multiply by 10; total bilirubin to micromoles per liter, multiply by 17.104; triglyceride to millimoles per liter, multiply by 0.0113.

^a^ In race, others were defined as combined categories of Asian, American Indian or Alaska Native, Native Hawaiian, or other Pacific Islander.

^b^ Represents a standard difference of more than 0.1.

### Patient Characteristics

Table 1 describes the patient demographics, baseline comorbidities, laboratory parameters, and medications in both groups; most variables were similar in both groups (SMD, <0.1). However, there were some residual differences: patients who underwent BS, compared with those who did not, had a higher baseline BMI (mean [SD], 43.4 [4.97] vs 42.2 [5.26]; SMD, 0.2379) and systolic blood pressure (mean [SD], 132 [16.7] vs 129 [16.0]; SMD, 0.1964), whereas the non-BS group had a higher hemoglobin A_1c_ than the BS group (mean [SD], 6.48% [1.65%] vs 6.15% [1.22%]; SMD, 0.2253 [to convert to proportion of total hemoglobin, multiply by 0.01]). Patients in the non-BS group had higher mean values of alanine aminotransferases than those in the BS group (39.0 [37.8] U/L vs 35.4 [31.5] U/L; SMD 0.104 [to convert to microkatal per liters, multiply by 0.0167]), whereas other values for the liver chemistries were similar. Medication use was similar in both groups (Table 1).

### Outcomes

Mean (SD) follow-up was 5.1 (1.7) years for the BS group and 4.3 (1.1) years for the non-BS group. The BS group, compared with the non-BS group, had significantly lower risk of new-onset of HF (HR, 0.60; 95% CI, 0.51-0.70), composite cardiovascular events (HR, 0.53; 95% CI, 0.44-0.65), composite cerebrovascular events (HR, 0.59; 95% CI, 0.51-0.69), and composite coronary artery interventions (HR, 0.47; 95% CI, 0.35-0.63) (Table 2).

**Table 2.** Associations of BS with Cardiovascular Events and All-Cause Mortality

Outcome	Events, No.		HR (95% CI)
	BS group	Non-BS group	
New-onset heart failure, follow-up, y			
1	149	263	0.55 (0.45-0.68)
3	183	33	0.54 (0.45-0.64)
5	203	356	0.56 (0.47-0.66)
7	220	375	0.56 (0.48-0.67)
Composite cardiovascular events, follow-up, y^a^			
1	49	152	0.31 (0.22-0.43)
3	82	208	0.39 (0.30-0.50)
5	105	232	0.44 (0.35-0.55)
7	122	242	0.48 (0.38-0.60)
Composite cerebrovascular events, follow-up, y^b^			
1	104	204	0.50 (0.39-0.63)
3	150	298	0.49 (0.40-0.60)
5	181	344	0.51 (0.43-0.61)
7	212	365	0.55 (0.46-0.65)
Composite coronary artery interventions, follow-up, y^c^			
1	23	81	0.28 (0.17-0.44)
3	20	100	0.29 (0.19-0.44)
5	44	112	0.38 (0.27-0.55)
7	56	124	0.42 (0.31-0.58)
All-cause mortality, follow-up, y			
1	14	49	0.30 (0.17-0.54)
3	32	91	0.34 (0.23-0.52)
5	42	111	0.37 (0.26-0.53)
7	57	123	0.44 (0.32-0.60)

Abbreviations: BS, bariatric surgery; HR, hazard ratio.

^a^ Composite cardiovascular events were defined as the first occurrence of unstable angina, myocardial infarction, or revascularization, including percutaneous coronary intervention or coronary artery bypass graft.

^b^ Composite end point of cerebrovascular disease was defined as the first occurrence of stroke (ischemic or hemorrhagic stroke), cerebral infarction, transient ischemic attack, carotid intervention, or surgery.

^c^ Composite coronary artery interventions were based on the requirement for coronary stenting, percutaneous coronary intervention, or coronary artery bypass.

**Figure 2.**
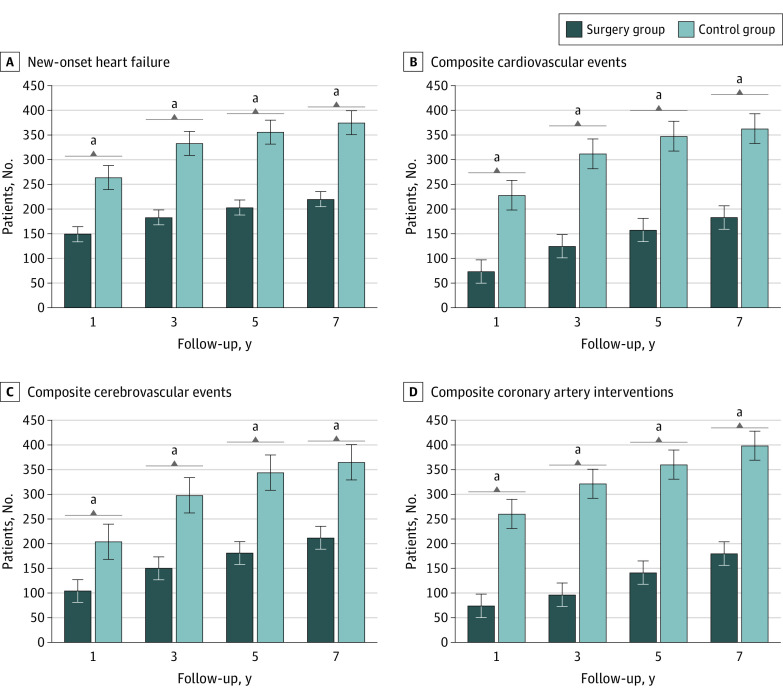
Incidence of Cardiovascular Disease in the Bariatric Surgery Group vs the Non–Bariatric Surgery Control Group Composite cardiovascular events were defined as the first occurrence of unstable angina, myocardial infarction, or revascularization, including percutaneous coronary intervention or coronary artery bypass graft; composite end point of cerebrovascular events, defined the first occurrence of stroke (ischemic or hemorrhagic stroke), cerebral infarction, transient ischemic attack, carotid intervention, or surgery; composite coronary artery interventions, based on the requirement for coronary stenting, percutaneous coronary intervention, or coronary artery bypass. ^a^Statistically significant difference.

The total number of patients who remained in follow-up was 3679 patients in the BS group and 3727 patients in the non-BS group at the end of 1 year, 2092 patients in the BS group and 2133 patients at the end of 3 years, 1056 patients in the BS group and 1069 patients in the non-BS group at the end of 5 years, and 769 patients in the BS group and 516 patients in the non-BS group at the end of 7 years. Rates of incidence of new-onset HF were significantly lower in the BS group than in the matched non-BS control group (Table 2 and Figure 2). Similarly, patients in the BS group had a significantly lower risk of incident composite cardiovascular events, cerebrovascular events, and coronary artery interventions than matched non-BS patients (Figure 2).

Similarly, the cumulative incidence of new onset of HF at 7 years was significantly lower in the BS group (HR, 0.56; 95% CI, 0.48-0.67) (Table 2 and Figure 2). BS was significantly associated with a lower hazard of reaching the other primary outcome, incident cardiovascular events (HR, 0.48; 95% CI, 0.38-0.60), composite cerebrovascular events (HR, 0.55; 95% CI, 0.46-0.65), and a composite outcome of coronary artery interventions (HR, 0.42; 95% CI, 0.31-0.58) (Table 2). Kaplan-Meier survival analysis showed that the cumulative probability of being event-free up to 7 years from the index event remained significantly lower in the non-BS group compared with the BS group for all studied outcomes (log-rank *P* < .001) (eFigure 2 in Supplement 1).

### All-Cause Mortality

**Figure 3.**
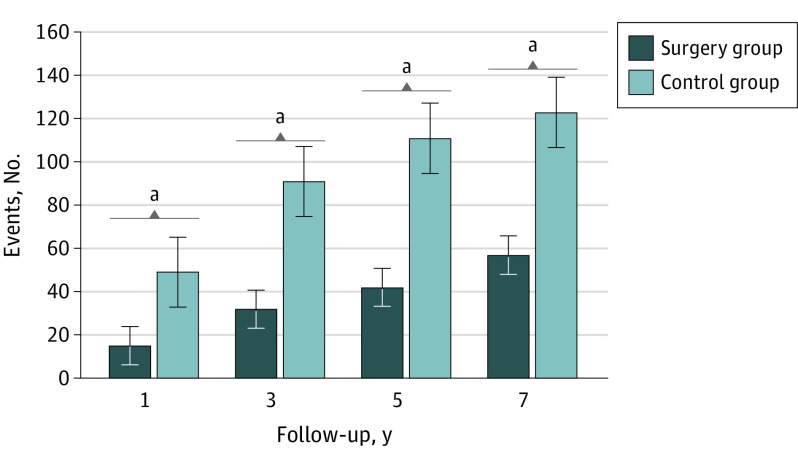
Incidence of All-Cause Mortality in the Bariatric Surgery Group vs the Non–Bariatric Surgical Control Group ^a^Statistically significant difference.

Mortality was significantly lower in the BS group than the non-BS group (HR, 0.56; 95% CI, 0.42-0.74). Risks of 1-, 3-, 5- and 7-year all-cause mortality were also significantly lower in the BS group than the matched non-BS controls (Table 2 and Figure 3). Kaplan-Maier survival analyses also revealed worse survival in the non-BS group compared with the BS group (log-rank *P* < .001) (eFigure 3 in Supplement 1).

### Sensitivity Analysis

Results of the sensitivity analyses are provided in eTable 1 in Supplement 1. Results of the sensitivity analysis were consistent with the results from the primary study analysis, and all statistically significant associations remained unchanged.

### Secondary Analysis

In the secondary analysis comparing CVD outcomes between patients with obesity without NAFLD who underwent BS and patients with obesity without NAFLD who did not undergo BS, BS was associated with a reduction in CVD outcomes, including risk of new-onset of HF (HR, 0.40; 95% CI, 0.37-0.45), composite cardiovascular events (HR, 0.52; 95% CI, 0.46-0.60), composite cerebrovascular events (HR, 0.54; 95% CI, 0.49-0.60), and composite coronary artery interventions or surgical treatments (HR, 0.44; 95% CI, 0.36-0.53). Similar findings were observed in secondary outcome mortality (HR, 0.41; 95% CI, 0.35-0.47) (eTable 2 in Supplement 1).

### Postoperative Complications After BS

Within 30 days after bariatric surgery, 271 patients (5.8%) experienced postoperative complications. Complications included postprocedural hemorrhage (51 patients [1.1%]), gastrointestinal leak (61 patients [1.3%]), postoperative sepsis (58 patients [1.2%]), venous thromboembolism (19 patients [0.4%]), small bowel obstruction (36 patients [0.8%]), acute postprocedural respiratory failure (12 patients [0.2%]), and acute kidney injury (53 patients [1.1%]) (eTable 3 in Supplement 1).

## Discussion

This was a large population-based, retrospective cohort study analysis that used a large sample size from a nationally representative database, statistical adjustments with a priori–identified potential confounders to balance the critical variables at the baseline by PSM, and a long duration of follow-up (7 years). Results indicate that BS was associated with decreased risk of major adverse cardiovascular events; new-onset of HF; cardiovascular events, such as unstable angina, myocardial infarction, cerebrovascular events; coronary artery interventions; and mortality. These outcomes were studied at various follow-up intervals, and consistent results were obtained at 1, 3, 5, and 7 years of follow-up.

Our findings strengthen previously reported associations and add novel data to the current literature. BS has already been reported to be associated with improved long-term adverse cardiovascular outcome risk in patients with obesity and diabetes in matched observational studies.^13,14^ However, there is a paucity of data on the associations of BS with cardiovascular events and mortality in patients with NAFLD. A 2021 cohort study by Aminian et al^15^ was the first study, to our knowledge, to observe a lower risk of major adverse cardiovascular events in their patients with biopsy-proven nonalcoholic steatohepatitis (NASH) without cirrhosis.^15^ However, individual analysis of the components of the major cardiovascular events was not defined because of the small number of events, and mortality was not studied as an outcome. Notably, the findings of our large, matched cohort study suggest that BS was associated with a lower risk of all components of major adverse cardiovascular events individually and overall all-cause mortality during long-term follow-up. Moreover, the study by Aminian et al^15^ only included patients with fibrotic NASH, and these findings cannot be generalizable to the broader NAFLD population.^12^ Thus, the association of BS with improved CVD outcomes and mortality in an overall NAFLD population noted in our study is a novel finding. Furthermore, our analysis adds new data regarding the association of a therapeutic modality with NAFLD outcomes beyond the surrogate end points of histologic improvement or regression. The improvement in mortality observed in the BS cohort was previously unreported for any NAFLD subset, to our knowledge, and supported the use of BS as a therapeutic agent to improve clinical outcomes in NAFLD.

The elevated incidences of CVDs and mortality risk in patients with NAFLD observed in this study likely derive from the liver- and NASH-specific increase in adverse cardiovascular outcomes, as well as the other comorbid diseases that these patients tend to have. Obesity is the primary etiologic contributor to NAFLD: it develops and progresses NAFLD by fat accumulation, development of insulin resistance, activation of the immune system, and alteration in the neurohormonal state.^16^ BS is the most effective obesity treatment; it reduces accumulated fat and improves peripheral insulin resistance with weight loss.^17^ On the other hand, BS has also been shown to histologically improve NAFLD and NASH and reduce liver-related adverse outcomes. A study by Lassaily et al^18^ found histologic resolution of NASH in 84% of patients 5 years after BS. A lower risk of progression to major liver-related adverse outcomes in patients with NASH who underwent BS compared with nonsurgical controls has been recently noted.^15^ Thus, BS can be theorized to mitigate the risk of adverse cardiovascular outcomes in these patients by improving comorbidities as well as NAFLD itself. BS improves both the liver histology (NAFLD/NASH process) and the overall nonliver cardiometabolic risk factor profile.^19,20^ Thus, it is not surprising that our study noted improved adverse cardiovascular events as well as mortality in patients with NAFLD who underwent BS.

In a secondary analysis, we compared CVD outcomes between patients with obesity without NAFLD who underwent BS and patients with obesity without NAFLD who did not undergo BS. Consistent with prior scientific data,^21,22^ BS was associated with a reduction in adverse CVD outcomes in this comparison. The HRs were lower than those for the NAFLD cohort comparisons (primary study outcomes). These data suggest potentially lower mitigation in adverse CVD outcomes after BS in patients with NAFLD compared with patients without NAFLD with obesity undergoing BS. However, direct comparison through clinical trials and prospective comparative studies is needed to draw definitive conclusions. Patients with NAFLD constitute a higher CVD–risk cohort, and it is possible that the CVD risk reduction in this cohort by BS is secondary to obesity-related CVD risk mitigation without significant reduction in the obesity-independent CVD risk posed by NAFLD.

### Strengths and Limitations

This study has several strengths. First, we included a robust control and adjustment for baseline and potential confounders. Second, the large sample in the propensity-matched analyses resulted in narrow CIs. It allowed us to capture a significant number of major adverse cardiovascular events and mortality incidents, which lends strength to the conclusions derived. Third, we performed a sensitivity analysis, and the statistically significant associations remained unchanged. Additionally, we performed a secondary analysis comparing CVD outcomes and mortality between patients with obesity without NAFLD who underwent BS and patients with obesity without NAFLD who did not undergo BS.

Our study also has some limitations. Data derived from EHR-based databases are susceptible to errors in coding or data entry, including misclassification and incomplete documentation. However, care was taken to use standardized measures to identify outcomes and minimize documentation errors. In addition, we have used an American Association for the Study of Liver Diseases–proposed validated diagnostic algorithm to identify individuals with NAFLD.^23^ Second, although we adjusted our analyses, some residual confounding is still possible. Third, our data did not include imaging modalities (eg, conventional imaging techniques or elastography) to confirm the diagnosis of NAFLD. Severe and progressive forms of NAFLD (eg, NASH) or surrogate serum markers for fibrosis (eg, NAFLD fibrosis score, Fibrosis-4 Index, and alanine aminotransferase to aspartate aminotransferase ratio) were not evaluated. In addition, the inclusion criteria were not based on histologic diagnosis and are thus susceptible to misdiagnosis. Fourth, we did not assess outcome differences between the different metabolic surgical procedures, namely RYGB or SG. Fifth, we could not determine the association between the surgical procedure and CVD by disease phenotype because of the lack of reliable noninvasive imaging modalities for NAFLD. Similarly, we did not grade the severity of comorbid conditions at baseline, which can also result in some selection bias. However, we believe these limitations represent a minor challenge to the study’s conclusions.

## Conclusions

The findings of this cohort study suggest that BS was associated with a lower incidence of major adverse cardiovascular events and all-cause mortality in patients with NAFLD and obesity. Although our study provides novel information, randomized clinical trials and additional observational studies are needed to corroborate our findings.

## Notes

Supplement 1. **eAppendix.** Supplementary Methods **eTable 1. **Sensitivity Analysis Measuring Outcomes Associated With Bariatric Surgery vs No Bariatric Surgery in Patients With Obesity and Nonalcoholic Fatty Liver Disease After Excluding Index Events Within 2 Years After the Index Date **eTable 2.** Secondary Analysis Measuring Outcomes From the Initial Time of Diagnosis of Nonalcoholic Fatty Liver Disease as the Index Event **eTable 3.** Postoperative Complications Within 30 Days After Bariatric Surgery **eFigure 1.** Propensity Score Density Graph for Patients With Nonalcoholic Fatty Liver Disease and Obesity Who Underwent Bariatric Surgery vs Controls Who Did Not Before and After Propensity Score Matching **eFigure 2.** Kaplan-Meier Curve Compares Cardiovascular Diseases in Patients Who Underwent Bariatric Surgery vs Controls Who Did Not **eFigure 3.** Kaplan-Meier Curve Compares Mortality in Patients Who Underwent Bariatric Surgery vs Control Who Did Not

Supplement 2.
Data Sharing Statement
