# Supplementary material for: Cardiovascular Outcomes and Mortality After Bariatric Surgery in Patients With Nonalcoholic Fatty Liver Disease and Obesity
Source: JAMA Netw Open. 2023 Apr 7;6(4):e237188. doi: 10.1001/jamanetworkopen.2023.7188 (PMC10082402; doi:10.1001/jamanetworkopen.2023.7188)
Supplement: Supplement 1. — eAppendix. Supplementary Methods eTable 1. Sensitivity Analysis Measuring Outcomes Associated With Bariatric Surgery vs No Bariatric Surgery in Patients With Obesity and Nonalcoholic Fatty Liver Disease After Excluding Index Events Within 2 Years After the Index Date eTable 2. Secondary Analysis Measuring Outcomes From the Initial Time of Diagnosis of Nonalcoholic Fatty Liver Disease as the Index Event eTable 3. Postoperative Complications Within 30 Days After Bariatric Surgery eFigure 1. Propensity Score Density Graph for Patients With Nonalcoholic Fatty Liver Disease and Obesity Who Underwent Bariatric Surgery vs Controls Who Did Not Before and After Propensity Score Matching eFigure 2. Kaplan-Meier Curve Compares Cardiovascular Diseases in Patients Who Underwent Bariatric Surgery vs Controls Who Did Not eFigure 3. Kaplan-Meier Curve Compares Mortality in Patients Who Underwent Bariatric Surgery vs Control Who Did Not [file jamanetwopen-e237188-s001.pdf]

# Supplemental Online Content

Krishnan A, Hadi Y, Alqahtani SA, et al. Cardiovascular outcomes and mortality after bariatric surgery in patients with nonalcoholic fatty liver disease and obesity. *JAMA Netw Open*. 2023;6(4):e237188. doi:10.1001/jamanetworkopen.2023.7188

**eAppendix.** Supplementary Methods

**eTable 1.** Sensitivity Analysis Measuring Outcomes Associated With Bariatric Surgery vs No Bariatric Surgery in Patients With Obesity and Nonalcoholic Fatty Liver Disease After Excluding Index Events Within 2 Years After the Index Date

**eTable 2.** Secondary Analysis Measuring Outcomes From the Initial Time of Diagnosis of Nonalcoholic Fatty Liver Disease as the Index Event

**eTable 3.** Postoperative Complications Within 30 Days After Bariatric Surgery

**eFigure 1.** Propensity Score Density Graph for Patients With Nonalcoholic Fatty Liver Disease and Obesity Who Underwent Bariatric Surgery vs Controls Who Did Not Before and After Propensity Score Matching

**eFigure 2.** Kaplan-Meier Curve Compares Cardiovascular Diseases in Patients Who Underwent Bariatric Surgery vs Controls Who Did Not

**eFigure 3.** Kaplan-Meier Curve Compares Mortality in Patients Who Underwent Bariatric Surgery vs Control Who Did Not

This supplemental material has been provided by the authors to give readers additional information about their work.

**eAppendix.** Supplementary Methods

**Data Source**

We used TriNetX (Cambridge, MA, USA), a global federated health research network providing real-time access to electronic health records (EHRs) from 56 healthcare organizations (HCOs) predominantly in the United States. The Real-time access to health insurance portability and accountability act– de-identified, compliant, and longitudinal clinical data to member HCOs is provided cloud-based. The de-identified clinical data, such as diagnoses, procedures, medications, laboratory values, and genomic information, are continuously aggregated directly from the EHR of the participating HCOs. Participating HCOs include outpatient, inpatient, and specialty care services and provide care to a diverse patient population from different ethnicity, age groups, geographical region, and income levels. Both the patients and HCOs, as data sources, stay anonymous.

As a federated network, TriNetX data have been granted a waiver from the Western institutional review board since only aggregated counts and statistical summaries of de-identified information without any protected health information were received from participating HCOs. In addition, no study-specific activities are performed in retrospective analyses.

**Standardizing the terminology and data quality check:**

The TriNetX software verifies the basic formatting to confirm that data are appropriately characterized. Patient counts were rounded up to the nearest 10 in our analysis to safeguard protected health information. TriNetX has production capabilities that have been tested that map data extensively from each of these structures to the standard model within TriNetX and can extract details of interest from the narrative content of clinical documents using natural language processing. TriNetX enforces a list of required fields (e.g., patient identifier) and rejects those records where the required data is lacking. Referential integrity checking confirms that data spanning multiple database tables can be successfully joined together. TriNetX requires at least 1 non-demographic fact for a patient to be calculated in a given

© 2023 Krishnan A et al. *JAMA Network Open.*

data set. Patient records with only demographic information are not included in data sets. As the data are refreshed, the TriNetX software monitors change in data volumes over time to ensure data validity.

**eMethod 2. Selection of Patients:**

The search was conducted following the criteria provided by TriNetX to identify potential patients. These codes included the International Classification of Diseases, Tenth Revisions, and Clinical Modification (ICD-10 CM). We combined patients into a single cohort of NAFLD/NASH defined by a diagnosis of NAFLD based on ICD-10-CM. (K76.0, and K75.81)

**Exclusion criteria:**

We excluded the patients if they had a diagnosis of another defined cause of liver disease other than NAFLD, including acute alcohol abuse or chronic alcohol abuse, alcoholic liver disease, toxic liver disease, viral hepatitis, Wilson's disease, autoimmune hepatitis, Gaucher disease, primary biliary cholangitis, hemochromatosis, primary sclerosing cholangitis. Diagnosis codes based on the exclusion criteria are listed below. K72 Acute and subacute hepatic failure without coma necrosis; K76.2 Central hemorrhagic necrosis of liver necrosis; K700 Alcoholic fatty liver hepatitis; K7010 Alcoholic hepatitis without ascites hepatitis; K7030 Alcoholic cirrhosis. Of liver without ascites cirrhosis. K709 Alcoholic liver disease, unspecified cirrhosis; K730 Chronic persistent hepatitis, not elsewhere classified hepatitis; K732 Chronic active hepatitis, not elsewhere classified hepatitis; K738 Other chronic hepatitis, not elsewhere classified hepatitis; K739 Chronic hepatitis, unspecified hepatitis; K740 Hepatic fibrosis; K74.1 Hepatic sclerosis cirrhosis; K743 Primary biliary cirrhosis;K74.4 Secondary biliary cirrhosis;K74.5 Biliary cirrhosis, unspecified cirrhosis;K74.60 Unspecified cirrhosis of liver cirrhosis;K74.69 Other cirrhosis of liver cirrhosis;K75.4 Autoimmune hepatitis;K76.89 Other specified diseases of liver cirrhosis;K76.9 Liver disease, unspecified cirrhosis;K76.7 Hepatorenal

© 2023 Krishnan A et al. *JAMA Network Open.*

syndrome cirrhosis;K77 Liver disorders in diseases classified elsewhere hepatitis;K71.6 Toxic liver disease with hepatitis, not elsewhere classified hepatitis;B17.0 Acute delta-(super) infection of hepatitis B carrier hepatitis;B1710 Acute hepatitis C without hepatic coma hepatitis;B17.2 Acute hepatitis E hepatitis;B17.8 Other specified acute viral hepatitis;B18.2 Chronic viral hepatitis C hepatitis;B18.8 Other chronic viral hepatitis;B18.9 Chronic viral hepatitis, unspecified hepatitis;B0081 Herpesviral hepatitis hepatitis;B15.0 Hepatitis A with hepatic coma hepatitis;B15.9 Hepatitis A without hepatic coma hepatitis;B16.0 Acute hepatitis B with delta-agent with hepatic coma hepatitis;B16.1 Acute hepatitis B with delta-agent without hepatic coma hepatitis;B16.2 Acute hepatitis B without delta-agent with hepatic coma hepatitis;B16.9 Acute hepatitis B without delta-agent and without hepatic coma hepatitis;B17.11 Acute hepatitis C with hepatic coma hepatitis;B17.2 Acute hepatitis E hepatitis;B17.8 Other specified acute viral hepatitis;B179 Acute viral hepatitis, unspecified hepatitis;B18.0 Chronic viral hepatitis B with delta-agent hepatitis;B18.1 Chronic viral hepatitis B without delta-agent hepatitis;B18.2 Chronic viral hepatitis C hepatitis;B19.0 Unspecified viral hepatitis with hepatic coma hepatitis;B19.10 Unspecified viral hepatitis B without hepatic coma hepatitis;B19.11 Unspecified viral hepatitis B with hepatic coma hepatitis; B19.20 Unspecified viral hepatitis C without hepatic coma hepatitis; B19.21 Unspecified viral hepatitis C with hepatic coma hepatitis; B19.9 Unspecified viral hepatitis without hepatic coma hepatitis; B25.1 Cytomegaloviral hepatitis; B25.1 Cytomegaloviral hepatitis; B26.81 Mumps hepatitis; B58.1 Toxoplasma hepatitis; B942 Sequelae of viral hepatitis; K70.11 Alcoholic hepatitis with ascites hepatitis; E83.01, Wilson's disease; K76.9 Liver disease, unspecified hepatitis; K72.90 Hepatic failure, unspecified without coma cirrhosis; K73.1 Chronic lobular hepatitis, not elsewhere classified hepatitis; K75.2 Nonspecific reactive hepatitis; K75.3 Granulomatous hepatitis, not elsewhere classified hepatitis;

K75.89 Other specified inflammatory liver diseases hepatitis; K70.31 Alcoholic cirrhosis of liver with ascites cirrhosis; F10.2 Alcohol dependence; K71.7 Toxic liver disease with fibrosis and cirrhosis of the liver.

**eMethod 3. Procedure codes to assist in identifying types of bariatric surgical procedures:**

All patients who underwent bariatric surgery [Roux-en-Y gastric bypass (RYGB) or sleeve gastrectomy (SG)] were included in the analysis.

Current procedural terminology codes: 43775, 43775 laparoscopic SG; 43843 (possible SG); 43644, 43645, 43844, 43846, 43847, 43842: Roux-en-Y gastric bypass; 43659: Laparoscopic Procedures on the Stomach (can be RYGB or SG); CPT: 43644, 43645; procedure Code: 0D190Z9, 0DB60ZZ, 0DB80ZZ, 0D16479, 0D1647A, 0D164J9, 0D164JA, 0D164K9, 0D164KA.

Diagnosis and healthcare common procedure coding system code (HCPCS): 43.82, Excision of the stomach, percutaneous endoscopic approach, vertical (SG); 44.31, 43.89, 44.38, 44.39: RYGB; S2085 Laparoscopic gastric bypass (RYGB); procedure Code: 0DB60ZZ; CPT: 43775.

**Diagnosis and procedure codes to assist in identifying cardiovascular conditions:**

Coronary artery disease: I25, I259; Acute myocardial infarction: ICD: i21.X and i21.XX, i22.X and i22.XX, i23.X and i23.XX; Stroke: i63.X and i63.XX and i63.XXX; Heart failure: I50.X; I50.XX; I50. XXX; Cerebrovascular event: Diagnoses and procedure: 433·X1, 434·X1, 436·0, 430·X, 431·X, I67.XX, I60.X; 38·12, 0·61, 0·63, CPT: 37215, 37216, 0075T, 0076T, 35301, 37205, 37206; Coronary artery disease: ICD for diagnose and procedure: 410.X, 411.X, 411.X AND 414.X, 36.01, 36.02, 36.03, 36.05, 36.06, 36.07, 36.10, 36.11, 36.12, 36.13, 36.14, 36.15, 36.16, 36.17, 36.19, 36.31, 36.32, 36.33, 36.64; CPT: 92982, 92984, 92995, 92996, 92980,

© 2023 Krishnan A et al. *JAMA Network Open.*

92981, 33510, 33511,33512, 33513, 33514, 33516, 33517, 33518, 33519, 33521, 33522, 33523, 33530, 33533, 33534, 33535, 33536, 93539, 9354

© 2023 Krishnan A et al. *JAMA Network Open.*

**eTable 1.** Sensitivity Analysis Measuring Outcomes Associated With Bariatric Surgery vs No Bariatric Surgery in Patients With Obesity and Nonalcoholic Fatty Liver Disease After Excluding Index Events Within 2 Years After the Index Date

| **Outcomes** | **BS** (N=4687) Events, n | **Non-BS** (N=4687) Events, n | **Hazard Ratio (95% CI)** |
|---|---|---|---|
| **At 7 years (after excluding a year from the index date)** | | | |
| **Primary outcome** | | | |
| New-onset HF | 157 | 246 | 0.61(0.50-0.75) |
| Composite cardiovascular events[a] | 95 | 133 | 0.68(0.52-0.88) |
| Composite cerebrovascular events[b] | 162 | 252 | 0.60(0.49-0.74) |
| Coronary artery interventions or surgeries[c] | 43 | 86 | 0.47(0.32-0.67) |
| **Secondary outcome** | | | |
| All-cause mortality | 42 | 76 | 0.51(0.35-0.75) |
| **At 7 years (after excluding 2 years from the index date)** | | | |
| **Primary outcome** | | | |
| New-onset HF | 131 | 185 | 0.64(0.53-0.84) |
| Composite cardiovascular events[a] | 83 | 96 | 0.81(0.61-0.98) |
| Composite cerebrovascular events[b] | 129 | 190 | 0.63(0.50-0.79) |
| Coronary artery interventions or surgeries[c] | 40 | 65 | 0.57(0.38-0.84) |
| **Secondary outcome** | | | |
| All-cause mortality | 32 | 51 | 0.57(0.36-0.89) |

**Abbreviations**: BS, bariatric surgery; HF, heart failure.
[a] Composite cardiovascular events were defined as the first occurrence of unstable angina, myocardial infarction, or revascularization, including percutaneous coronary intervention or coronary artery bypass graft.
[b]A composite endpoint of cerebrovascular events was defined as the first occurrence of stroke (ischemic or hemorrhagic stroke), cerebral infarction, transient ischemic attack, carotid intervention, or surgery.
[c]A composite endpoint of coronary artery interventions and surgeries were defied based on patients who had any interventions/surgeries after BS (coronary angioplasty or stents, percutaneous coronary intervention, or coronary artery bypass).

© 2023 Krishnan A et al. *JAMA Network Open.*

**eTable 2.** Secondary Analysis Measuring Outcomes From the Initial Time of Diagnosis of Nonalcoholic Fatty Liver Disease as the Index Event

| **Outcomes** | **BS** (N=4687) Events, n | **Non-BS** (N=4687) Events, n | **Hazard Ratio (95% CI)** |
|---|---|---|---|
| **Primary outcome** | | | |
| New-onset HF | 167 | 217 | 0.61(0.50-0.75) |
| Composite cardiovascular events[a] | 402 | 437 | 0.68(0.52-0.88) |
| Composite cerebrovascular events[b] | 197 | 221 | 0.60(0.49-0.74) |
| Coronary artery interventions or surgeries[c] | 47 | 78 | 0.47(0.32-0.67) |
| **Secondary outcome** | | | |
| All-cause mortality | 51 | 64 | 0.51(0.35-0.75) |

**Abbreviations**: BS, bariatric surgery; HF, heart failure.
[a] Composite cardiovascular events were defined as the first occurrence of unstable angina, myocardial infarction, or revascularization, including percutaneous coronary intervention or coronary artery bypass graft.
[b]A composite endpoint of cerebrovascular events was defined as the first occurrence of stroke (ischemic or hemorrhagic stroke), cerebral infarction, transient ischemic attack, carotid intervention, or surgery.
[c]A composite endpoint of coronary artery interventions and surgeries were defied based on patients who had any interventions/surgeries after BS (coronary angioplasty or stents, percutaneous coronary intervention, or coronary artery bypass).

© 2023 Krishnan A et al. *JAMA Network Open.*

**eTable 3.** Postoperative Complications Within 30 Days After Bariatric Surgery

| **Postoperative complications** | **Total number of patient** (N= 271), Events, n (%) |
|---|---|
| Postprocedural hemorrhage | 51 (1.1) |
| Gastrointestinal leak | 61 (1.3) |
| Postoperative sepsis | 58 (1.2) |
| Venous thromboembolism | 19 (0.4) |
| Small bowel obstruction | 36 (0.8) |
| Acute postprocedural respiratory failure | 12 (0.2) |
| Acute kidney injury | 53 (1.1) |

**eFigure 1.** Propensity Score Density Graph for Patients With Nonalcoholic Fatty Liver Disease and Obesity Who Underwent Bariatric Surgery vs Controls Who Did Not Before and After Propensity Score Matching

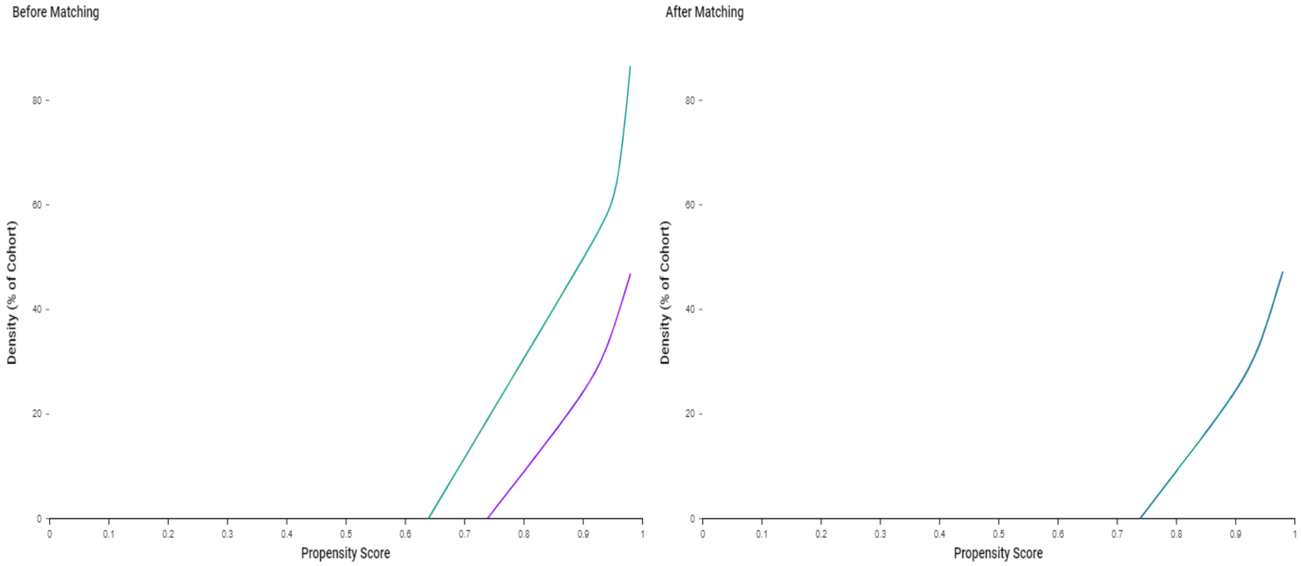


© 2023 Krishnan A et al. *JAMA Network Open.*

**eFigure 2.** Kaplan-Meier Curve Compares Cardiovascular Diseases in Patients Who Underwent Bariatric Surgery vs Controls Who Did Not

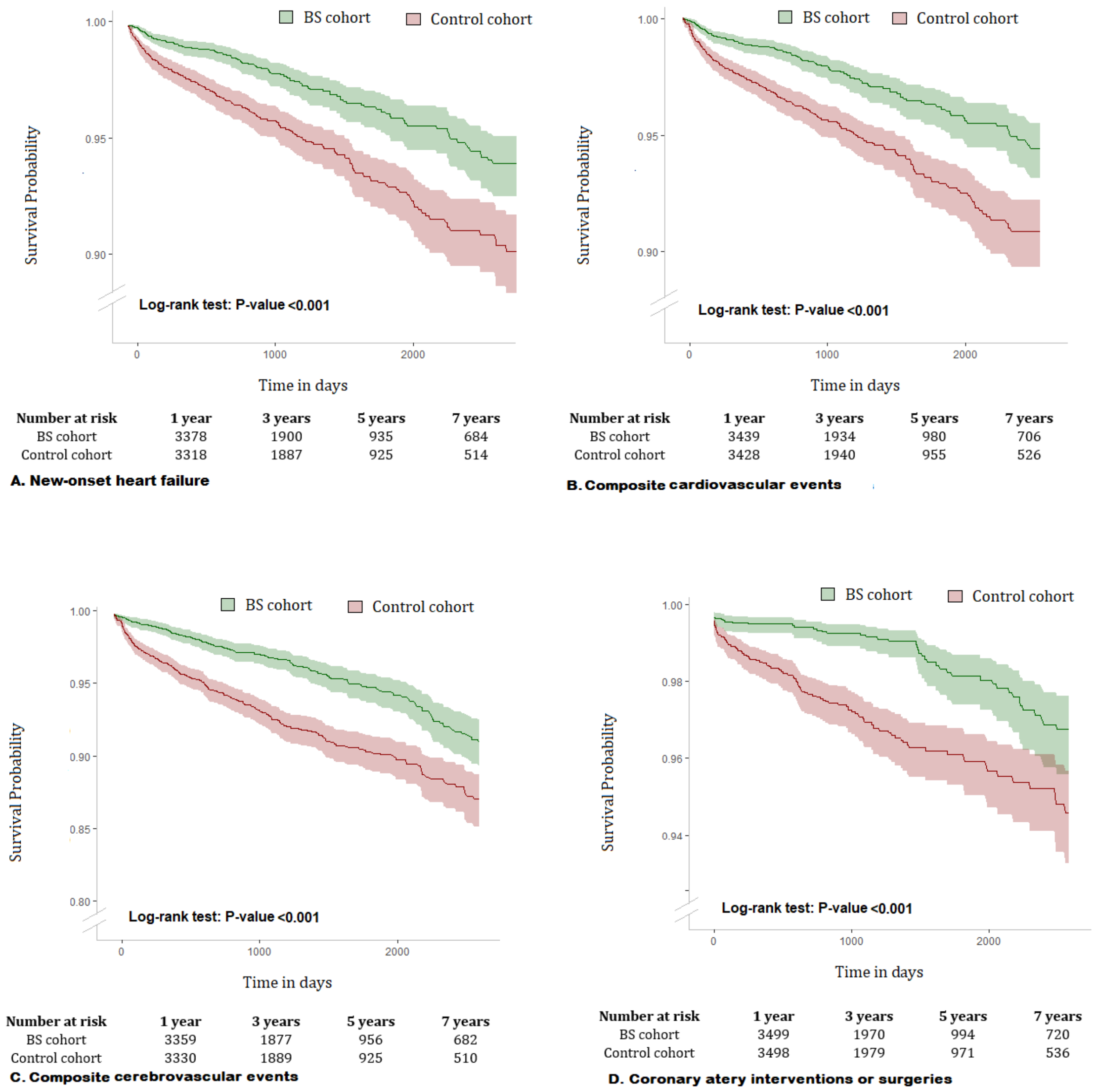


| Number at risk | 1 year | 3 years | 5 years | 7 years |
|---|---|---|---|---|
| BS cohort | 3378 | 1900 | 935 | 684 |
| Control cohort | 3318 | 1887 | 925 | 514 |

**A. New-onset heart failure**

| Number at risk | 1 year | 3 years | 5 years | 7 years |
|---|---|---|---|---|
| BS cohort | 3439 | 1934 | 980 | 706 |
| Control cohort | 3428 | 1940 | 955 | 526 |

**B. Composite cardiovascular events**

| Number at risk | 1 year | 3 years | 5 years | 7 years |
|---|---|---|---|---|
| BS cohort | 3359 | 1877 | 956 | 682 |
| Control cohort | 3330 | 1889 | 925 | 510 |

**C. Composite cerebrovascular events**

| Number at risk | 1 year | 3 years | 5 years | 7 years |
|---|---|---|---|---|
| BS cohort | 3499 | 1970 | 994 | 720 |
| Control cohort | 3498 | 1979 | 971 | 536 |

**D. Coronary atery interventions or surgeries**

Composite cardiovascular events were defined as the first occurrence of unstable angina, myocardial infarction, or revascularization, including percutaneous coronary intervention or coronary artery bypass graft. A composite endpoint of cerebrovascular disease was defined as the first occurrence of stroke

© 2023 Krishnan A et al. *JAMA Network Open.*

(ischemic or hemorrhagic stroke), cerebral infarction, transient ischemic attack, carotid intervention, or surgery. The composite coronary artery procedures or surgeries were based on the requirement for coronary stenting, percutaneous coronary intervention, or coronary artery bypass. Abbreviations: BS, bariatric surgery.

© 2023 Krishnan A et al. *JAMA Network Open.*

**eFigure 3.** Kaplan-Meier Curve Compares Mortality in Patients Who Underwent Bariatric Surgery vs Control Who Did Not

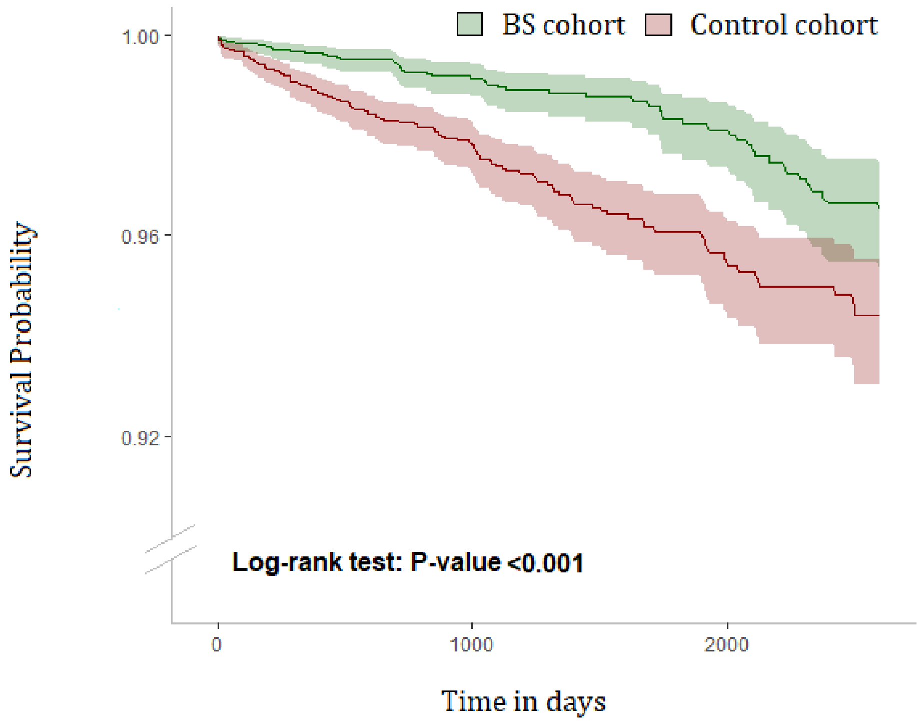


| Number at risk | 1 year | 3 years | 5 years | 7 years |
|---|---|---|---|---|
| BS cohort | 3488 | 1965 | 990 | 716 |
| Control cohort | 3478 | 1975 | 972 | 536 |

**eFigure 3. Supplementary Figure 3.** Kaplan–Meier curve compares mortality in patients who underwent bariatric surgery versus the non-bariatric surgical control group.

© 2023 Krishnan A et al. *JAMA Network Open.*
