# Supplementary material for: Cardiovascular Outcomes and Mortality After Bariatric Surgery in Patients With Nonalcoholic Fatty Liver Disease and Obesity
Source: JAMA Netw Open. 2023 Apr 7;6(4):e237188. doi: 10.1001/jamanetworkopen.2023.7188 (PMC10082402; doi:10.1001/jamanetworkopen.2023.7188)
Supplement: Supplement 2. — Data Sharing Statement [file jamanetwopen-e237188-s002.pdf]

Data Sharing Statement

Krishnan. Cardiovascular Outcomes and Mortality in After Bariatric Surgery in Patients With Nonalcoholic Fatty Liver Disease and Obesity. *JAMA Netw Open*. Published April 07, 2023. doi:10.1001/jamanetworkopen.2023.7188

Data
Data available: No

Additional Information
Explanation for why data not available: No additional data is available.
